# Supplementary material for: Epidemiology of Chronic Hepatitis C in First Nations Populations in Canadian Prairie Provinces
Source: Pathogens. 2025 Jul 14;14(7):693. doi: 10.3390/pathogens14070693 (PMC12299096; doi:10.3390/pathogens14070693)
Supplement: Supplementary file 1 [file pathogens-14-00693-s001.zip › pathogens-3697835-supplementary.pdf]

Supplementary S1-S4

S1. Glossary

Child welfare system / Foster Care / 60's Scoop / Millennium Scoop: The current and past government run systems forcibly removing Indigenous children from their families, homes, and communities and placing them in non-Indigenous settings. Severing connection to culture, language and land and forcing assimilation and inter-generational trauma through discrimination, racism, neglect, physical, sexual, and emotional abuse [2].

.

Colonization: The acts of European entities rooted in religion, politics, economics and power used to subjugate and exploit Indigenous Peoples in the process of settling Canada [2].

Decolonize: The undoing or divesting of colonial power of culture, languages, education, health and bureaucracy [2].

.

Indian Act of 1876: Federal legislation regulating Indigenous People's ability to live and access land, status, education, health supports and other aspects of daily life. Numerous amendments, revisions and re-enactments since the original document but still continues to impact all aspects of life for Indigenous Peoples in what is now Canada [3].

Indigenous: Inclusive reference to the First Nations (Indigenous Peoples who are not Inuit or Métis), Inuit (Indigenous Peoples of the arctic and sub-arctic areas of Canada) and Métis (descendants of unions between Indigenous Peoples and European fur traders via ethnogenesis creating a unique cultural entity) within what is known today as Canada [2].

Intergenerational Trauma: Past and ongoing traumatic events related to the colonizing of Canada inform the conscious or unconscious transfer of thoughts, behaviours, coping mechanisms, and health status with Indigenous Peoples [2].

Knowledge Keeper: Trusted and respected Indigenous community member recognized for their experiences, the knowledge they have received, and their willingness to share these with others [2].

Reserve: Tracts of land set aside for use by First Nations, but the Crown or federal government retains title to this land. Historically specific rules controlled passage to and from these lands [3].

Residential School: Institutions set up by religious entities with government funding to forcibly house Indigenous children who were removed from their families, homes and communities and sometimes taken across the country. These schools took over 150,000 children age 5-16 years and focused on manual labor skills versus educational excellence. Over 6,000 children died or disappeared from these schools [3].

Two-Eyed Seeing: Approach to learning and understanding by seeing from one eye with Indigenous ways of knowing and from the other eye with Western ways of knowing [2]

Treaty: An agreement, recognized constitutionally between First Nation, Inuit or Métis and the government of Canada, outlining obligations in return for access to land [2,3].

Truth and Reconciliation Commission and Calls to Action: The commission (2008-2015) was an element of the settlement for residential school survivors, and their families and communities, and established 94 calls to action to be taken by the government as steps to rectify harms and work toward reconciliation between Indigenous and non-Indigenous Peoples of Canada [2].

S2. Map of Alberta, Saskatchewan, Manitoba (from L to R), showing First Nation reserves

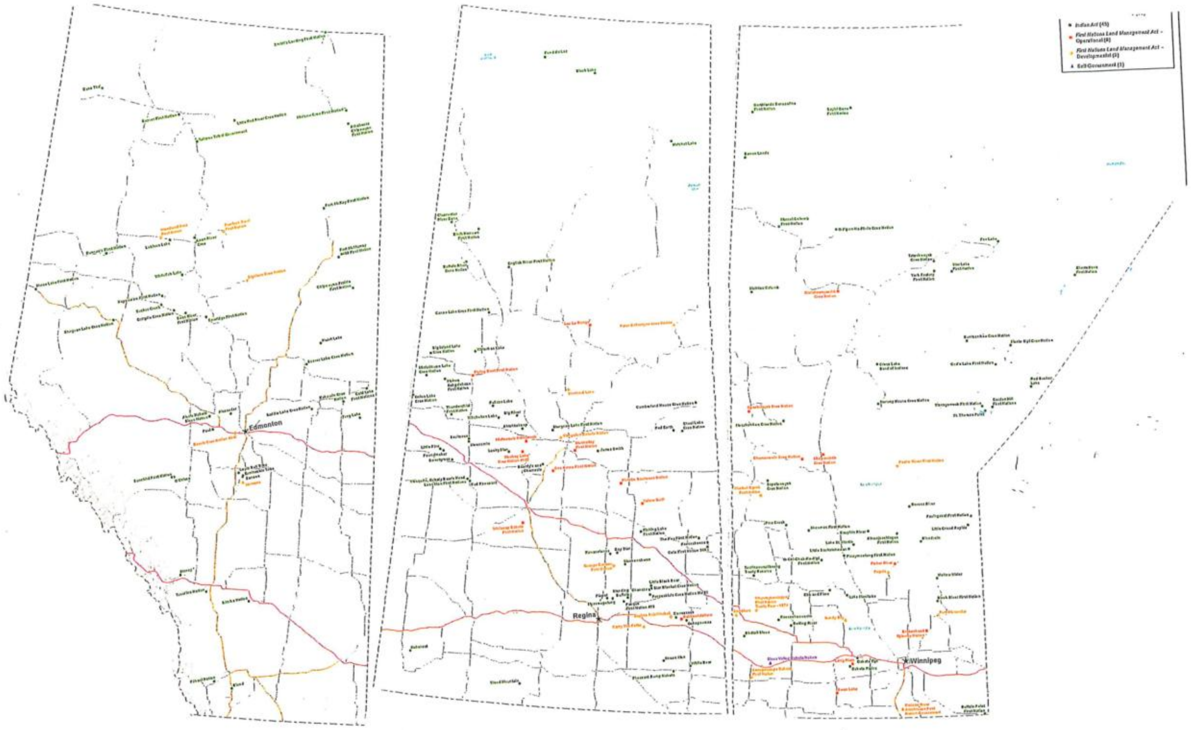

S3. Colonial context of hepatitis C

In the colonizing and settling phase of Canada's history over 3,400 reserved land locations were set aside to contain First Nations on what are now called reserves, where just over 37% of registered status First Nations people currently live [3]. Many of these reserves are located in remote areas thus limiting work opportunities, education supports and healthcare access. These gaps in equitable access in reserve communities may influence relocation by many First Nations

People to urban centres. First Nations, as co-signatories of the historical and modern treaties reside close to areas of natural resource extraction and are disproportionally affected by environmental degradation and social disruption resulting from the "boom and bust" business cycle, especially after companies maximize profits and move out of the area, often abandoning their responsibilities to support land rehabilitation and creation of sustainable regional development.

These disruptions to land are especially impactful for First Nations who are intimately connected to the land and rely on this connection for physical, mental, social and spiritual wellness. Patterns of subsistence lifestyle, family gatherings, ceremony and spiritual practice are intricately connected to the land and the seasonal cycles of gathering. Reciprocal relationship with natural resources, plant medicines and food sources of specific regions, along with trading routes and meeting points are shaped over generations of reading the land and living in respectful relationship to the land as to a close relative. These relationships and patterns were disrupted, First Nations were removed from traditional lands and the visible and invisible relationship to land-based self-determination and wellness begun in the early colonization era continues today.

Between 1960 and 2023, Canada's population has more than doubled, while the country's gross domestic product (GDP) increased more than 50-fold from about 40 billion to 2.1 trillion dollars [4]. Although this may be seen as growth, it comes at extensive cost to the continuance of the land and violation of the sovereignty of First Nations, the original stewards of the land. The economic development of such a magnitude has required mobilization of large segments of Canadian society and the country's resources. While this has increased an overall economic participation, it has exacerbated pre-existing socio-economic and income inequality and has come at an unsustainable and tremendous cost to the natural environment and the wellbeing of Indigenous populations.

This ongoing exploitation and colonization of land and its resources builds on the continued experience of colonial and assimilation-based legislation and policies. Starting with broken Treaties between First Nations and the Dominion of Canada, continuing with the Indian Act of 1876, evolving into forced attendance at residential schools and abusive control and abductions of First Nations children through the child welfare system, these actions have shaped the current lived experience for First Nations populations. This daily experience also includes discrimination, marginalization, disruption and transgenerational trauma while creating significant barriers in accessing education, employment, housing, food, security, healthcare services, clean water and sanitation.

These economic, legislative and population dynamics have inflicted a lasting disruption to the use of traditional languages, ways of learning, land-based diet and lifestyle, generational knowledge transfer, spirituality practices, coping strategies and the relational community circle previously experienced. This results in a loss of First Nations Ways of Being of a magnitude nearly impossible to quantify. While the vast and lasting harms of failed assimilation policies are becoming more broadly acknowledged in Canada, the nation-to-nation reconciliation process is still in its infancy.

Institutional racism in Canadian healthcare

Institutional or systemic racism can be defined as institutions-driven inequalities that are based on policies or practices that inherently discriminate – either deliberately or not - against one or more population groups [5]. In practice, systemic racism may be seen as a tendency by one social group to marginalize another social group in a form of exclusion from the application of the federal law [6] underfunding basic childcare services [7], creating obstacles to access health services [5] and otherwise subjecting it to a different standard of service[6]. Selective application of the law and law enforcement has been implicated in the excessive use of traffic stops of Indigenous Peoples in 2014-2017 [8] and disproportionate incarceration rates of Indigenous Peoples [9,10]. To the latter point, while Indigenous Peoples made up about 5% of the total

Canadian Population in 2022, they comprised approximately 32% of the total inmate population in Canada with even higher proportion of Indigenous women in-custody, at approximately 50% of all female inmates in the federal penitentiaries [9,10].

Canadian history presents examples of more subtle, but comparably harmful institutional racism policies and practices. One is how First Nations health data has been explicitly misused and appropriated throughout Canadian history to inform colonization processes and to sustain racist policies of subjugation [11]. By using the Indian Act and the limited representativeness and damaging depictions of First Nations in the health and social data they collected, government agencies and the Royal Canadian Mounted Police "pathologized and took action against [Indigenous communities] in a form of forceful removal of more than 150,000 Indigenous children from their families in the residential school system and the '60s scoop'"[12]. In examining how information about First Nations has been collected and used in the past, the Royal Commission on Aboriginal Peoples in Canada concluded that First Nations "have not been consulted about what information should be collected, who should gather that information, who should maintain it and who should have access to it" [13]. According to a recent report by the First Nations Information Governance Centre in Alberta, Canada, both "the context & purposes of data have historically been determined outside First Nations communities and the misuse of data has led to situations of misappropriation and broken trust" [11].

Some progress has been made but much more is needed. The publication of the 1996 Report of the Royal Commission on Aboriginal Peoples and the release in 2015 of the Calls to Action by the Truth and Reconciliation Commission [13,14], were established to document and inform Canadians about the experiences of First Nations who attended residential schools. The impact of racist colonial policies on the availability, accessibility, timeliness, accuracy and comprehensiveness of health outcome data in First Nations as some of the populations bearing the brunt of the disease burden continues today.

National and jurisdictional (i.e. provincial or territorial) level health reports rarely provide an insight into the burden of disease or treatment outcomes by ethnicity or Indigenous status and not being able to see themselves in the national or provincial/territorial data has been an on-going concern of First Nation organizations in Canada (FNIGC, First Nations Information Governance Centre, 2020, https://fnigc.ca). Furthermore, lack of disaggregated population-specific disease burden data continues to hamper community-led and community-specific efforts to achieve a meaningful reduction in the burden of nationally notifiable HCV infections. This continued gap calls for action to "redress the legacy of residential schools and advance the process of Canadian reconciliation" [14].

The immense loss and disruption experienced by First Nations through acts of colonization and assimilation, when dissociated from culture and relational support strategies, instead becomes a catalyst for coping behaviours which may be deemed harmful and or illegal. This cycle of loss, trauma and ongoing exposure to racism and further harms may exhibit as intergenerational or transgenerational trauma. Harm reduction is often used in reference to supply of clean needles, condoms, legally produced psychoactive substances and structures of medical supervision within a substance use context. But these medical approaches represent only a small part of many evolving distinctions-based culturally-grounded and community-led wholistic wellness activities and initiatives supporting healing for First Nations [15, 16, 17].

Unfortunately, harm reduction services in several Canadian jurisdictions have been curtailed despite evidence demonstrating positive impact and cost-effectiveness, as well as connecting persons who use substances to health supports, social services and treatment networks [18]. This approach will exacerbate the toxic drug crises and heighten inequities already experienced by First Nations in obtaining essential services [18]. Studies in Australia report harm reduction services specifically supporting needle and syringe programming prevented an estimated

96,000 HCV infections and over 32,000 human immunodeficiency virus (HIV) infections, thus saving four dollars in direct health care costs for every dollar invested in harm reducing needle syringe programs [19].

Harm reduction and wellness support activities may assist people in regaining their sense of self-worth, self-determination, and opportunities to re-engage in community. Pursuing individual and community wellness may include revitalization of First Nations languages, countering and preventing institutional and community racism, connecting to the land, traditional harm reduction practices and supporting awareness or prevention strategies. While HIV is still seen as the face of life-altering health outcomes with the potential to impact interactions between family and community, there is a growing appreciation of the similar life-altering impacts of HCV at the personal, family, community, and population levels. These impacts include stigma and self-stigma related to infection with a blood-borne, and / or sexually transmitted infectious disease often associated with substance use and multiple sexual partners, often generating fear and influencing ostracism from family and community. Although HIV may impact various body organs, chronic HCV infection can also impact other organs in addition to cirrhosis, liver cancer, liver failure and death.

S4. Regional definitions of HCV infection used were as follows:

- In MB: Manitoba Public Health Branch Communicable Disease Management Protocol
  - Laboratory-confirmed case–acute: Detection of HCV RNA or detection of HCV antigen (HCV Ag) AND clinical hepatitis (jaundice or peak elevated total bilirubin levels in serum ≥50 µmol/L or peak elevated serum alanine aminotransferase [ALT] >200 IU/L) within six months preceding the first positive HCV test AND negative Hepatitis A IgM antibody (anti-HAV IgM) and negative Hepatitis B core IgM antibody (anti-HBc IgM) AND No other known cause for clinical hepatitis; OR New detection of HCV antibodies (anti-HCV) or HCV RNA or HCV Ag in a patient with previously documented negative anti-HCV or negative HCV RNA within the preceding 12 months.
  - Note: Individuals who have achieved complete eradication of the virus, termed sustained virologic response (SVR) after treatment through documented undetectable HCV RNA at least 12 weeks post end-of-treatment (SVR-12), then have a subsequent detectable HCV RNA result within 12 months of SVR-12 date should be considered as having a new acute or recent infection for surveillance purposes, even though these cases may rarely represent late post-treatment relapses.
  - Laboratory-confirmed case–chronic: Does not meet criteria for acute or recent infection AND detection of HCV RNA; OR Detection of HCV Ag.
-
- In SK: Government of Saskatchewan Ministry of Health Communicable Disease Control Manual
  - Confirmed Case: Acute or Recent Infection: Detection of hepatitis C virus antibodies (anti-HCV) or hepatitis C virus RNA (HCV

RNA) in a person with discrete onset of any symptom or sign of acute viral hepatitis within 6 months preceding the first positive HCV test AND negative anti-HAV IgM, and negative anti-HBc IgM or HBsAg tests AND serum alanine aminotransferase (ALT) greater than 2.5 times the upper normal limit; OR Detection of hepatitis C virus antibodies (anti-HCV) in a person with a documented anti-HCV negative test within the preceding 12 months; OR Detection of hepatitis C virus RNA (HCV RNA) in a person with a documented HCV RNA negative test within the preceding 12 months.

- o Confirmed Case: Unspecified (including chronic and resolved infections): Detection of hepatitis C virus antibodies (anti-HCV); OR Detection of hepatitis C virus RNA (HCV RNA).
- o Note from region: the majority of cases are unspecified.

-
- In AB: Alberta Public Health Disease Management Guidelines – Acute Cases / Chronic Cases
  - o Confirmed Case: Acute or Recent Infection: Confirmed detection of hepatitis C virus (HCV) antibodies (anti-HCV) or hepatitis C virus RNA (HCV RNA) in a person with discrete onset of any symptom or sign of acute viral hepatitis within the previous 6 months of current positive test AND negative anti-HAV IgM and negative anti-HBc IgM or HBsAg test AND serum alanine aminotransferase (ALT) greater than 2.5 times the upper normal limit; OR Confirmed detection of hepatitis C virus antibodies (anti-HCV) or HCV RNA in a person with a documented anti-HCV negative test within the preceding 12 months; OR Detection of hepatitis C virus RNA (HCV RNA) in a person with a documented HCV RNA negative test within the preceding 12 months, excluding those undergoing HCV treatment or therapy; OR Individuals who have had a sustained virologic response (SVR) for six months post-treatment and become HCV RNA positive within 12 months of SVR should be considered as having an acute or recent infection for surveillance purposes, even though some of these cases may be post-treatment relapses.
  - o Confirmed Case: Chronic: Detection of anti-hepatitis C antibodies (anti-HCV) and should be confirmed by a second manufacturer's EIA, immunoblot or nucleic acid (e.g., PCR) for HCV-RNA; OR Detection of hepatitis C virus RNA (HCV-RNA).
  - o With AB Chief Medical Officer of Health approval: Died Blood Spot confirmatory testing from the National Micrbiology Laboratory (HCV antibody positive & HCV RNA positive)

Region-specific Data Limitations:

- In MB: Case data are sourced from Indigenous Services Canada Regional database and only include newly diagnosed cases of HCV. Population counts are sourced from Status Verification System (SVS). Although the SVS should be a complete list, there are often inaccuracies. For example, if births are not reported in a timely manner, the system will exclude a certain proportion of young children (0-4 years) who are entitled to Status but are currently covered by their

parents or remain unregistered for other reasons. Similarly, if deaths are not reported in a timely way, the system will include a certain proportion of deceased individuals. Residency is typically only updated when a life event is reported; therefore the on- and off-reserve designation may not be current. The community population counts for on-reserve will often be an under-estimation of the actual population being served. The on-reserve population count does not include any non-Status community members (i.e. First Nations who are not registered with Indigenous Services Canada and other members of the community who may not be full-time residents (ex. Royal Canadian Mounted Police, nursing staff, educators etc.) for whom the community provides health care services, nor any young children (0-4 years) living in the community who have not yet been registered with ISC.

- In SK: Case data are sourced from the provincial database.
- In AB: Case data are sourced from Indigenous Canada Regional Database and include only First Nations living in community.

References

2. Greenwood et al., . Introduction to Determinants of First Nation, Inuit and Métis Peoples' Health in Canada. Canadian Scholars, an imprint of CSP Books Inc. Toronto, ON, Canada, 2022.

3.. Joseph & Joseph. Indigenous Relations: Insights, tips and suggestions to make reconciliation a reality. Page Two Books, Inc. Vancouver, BC, Canada, 2019.

4. World Economics. Canada | GDP | 2021 | Economic Data [Internet]. World Economics. 2023. Available from: https://www.worldeconomics.com/Country-Size/canada.aspx
5. Souissi T. Systemic Racism in Canada | The Canadian Encyclopedia [Internet]. www.thecanadianencyclopedia.ca. 2022. Available from: https://www.thecanadianencyclopedia.ca/en/article/systemic-racism
6. Daniels v. Canada (Indian Affairs and Northern Development), 2016 SCC 12 (CanLII) [Internet]. Canlii.org. CanLII; 2016. Available from: https://www.canlii.org/en/ca/scc/doc/2016/2016scc12/2016scc12.html
7. https://s3.documentcloud.org/documents/2698184/Jugement.pdf
8. https://rapportspvm2019.ca/rapport/SPVM%20Stats_2019_ANG_FINAL.pdf
9. Government of Canada O of the AG of C. Report 4—Systemic Barriers—Correctional Service Canada [Internet]. www.oag-bvg.gc.ca. 2022. Available from: https://www.oag-bvg.gc.ca/internet/English/parl_oag_202205_04_e_44036.html
10. Over-representation of Indigenous persons in adult provincial custody, 2019/2020 and 2020/2021
11. Mcbride K. Data Resources and Challenges for First Nations Communities Document Review and Position Paper Prepared for the Alberta First Nations Information Governance Centre [Internet]. Available from: https://afnigc.ca/wp-content/uploads/2024/01/Data_Resources_Report.pdf
12. https://oci-bec.gc.ca/sites/default/files/2023-10/Annual%20Report%20EN%20%C3%94%C3%87%C3%B4%20Web.pdf.
13. The Report of the Royal Commission on Aboriginal Peoples (PRB 99-24E) [Internet]. publications.gc.ca. Available from: https://publications.gc.ca/Collection-R/LoPBdP/EB/prb9924-e.htm
14. Government of Canada. Truth and Reconciliation Commission of Canada: Calls to Action [Internet]. publications.gc.ca. 2015. Available from: https://publications.gc.ca/collections/collection_2015/trc/IR4-8-2015-eng.pdf
15. Pearce ME, Jongbloed K, Demerais L, et al. "Another thing to live for": supporting HCV treatment and cure among Indigenous people impacted by substance use in Canadian cities. International Journal of Drug Policy. 2019 Dec 1;74:52-61.
16. Pearce ME, Jongbloed K, Pooyak S, et al. The Cedar Project: exploring the role of colonial harms and childhood maltreatment on HIV and hepatitis C infection in a cohort study involving young Indigenous people who use drugs in two Canadian cities. BMJ open. 2021 Jul 1;11(7):e042545
17. Granfield R, Cloud W. Coming clean : overcoming addiction without treatment. New York: New York University Press; 1999.

18. Webster P. Canadian provinces scaling back harm-reduction services
Lancet 2024 Oct 19;404(10462):1509-1510.
19. National Centre in HIV Epidemiology and Clinical Research Return on investment 2: Evaluating the cost-effectiveness of needle and syringe programs in Australia [Internet]. 2009. Available from: https://www.acon.org.au/wp-content/uploads/2015/04/Evaluating-the-cost-effectiveness-of-NSP-in-Australia-2009.pdf
